# Biotransformation of Abiraterone Into Five Characteristic Metabolites by the Rat Gut Microbiota and Liver Microsomes

**DOI:** 10.3389/fonc.2022.890323

**Published:** 2022-07-22

**Authors:** Adili Keranmu, Fei-Ya Yang, Wasilijiang Wahafu, Su-Jun Han, Guo-Sheng Yang, Nian-Zeng Xing

**Affiliations:** ^1^ State Key Laboratory of Molecular Oncology, Department of Urology, National Cancer Center/National Clinical Research Center for Cancer/Cancer Hospital, Chinese Academy of Medical Sciences and Peking Union Medical College, Beijing, China; ^2^ Department of Urology, Shanghai East Hospital, School of Medicine, Tongji University, Shanghai, China; ^3^ Department of Urology, Shanxi Province Cancer Hospital/Shanxi Hospital Affiliated to Cancer Hospital, Chinese Academy of Medical Sciences/Cancer Hospital Affiliated to Shanxi Medical University, Taiyuan, Shanxi, China

**Keywords:** abiraterone, prostate cancer, gut microbiota, liver microsome, metabolites

## Abstract

It is well known that the role of gut microbiota in drug metabolism, especially in oral difficult absorbable drugs. Understanding the gut microbiota could enable us to understand drugs in new ways. The purpose of the study was to investigate explore the metabolites of the anti-prostate cancer drug Abiraterone by examining gut microbiota metabolism and hepatic metabolism *in vitro*. In this study, five metabolites (M1, M2, M3, M4 and M5) of Abiraterone were discovered using LC/MS^n^-IT-TOF. Four isomeric metabolites M1-M4 were found in liver microsome. M5 was found in the intestinal contents of Sprague-Dawley rats with a molecular weight of 388.31. Among them, M4 was found to be Abiraterone N-Oxide by comparison with the standard sample. After further comparing the metabolic behavior of Abiraterone in rat gut microbiota and liver microsomes, we delineated the possible metabolic pathways of Abiraterone. In conclusion, Abiraterone is metabolized specifically in liver microsomes and gut microbiota. This study can provide a theoretical basis for elucidating the metabolic mechanism of Abiraterone and guide its rational application in clinic.

## Introduction

**Figure 1:**
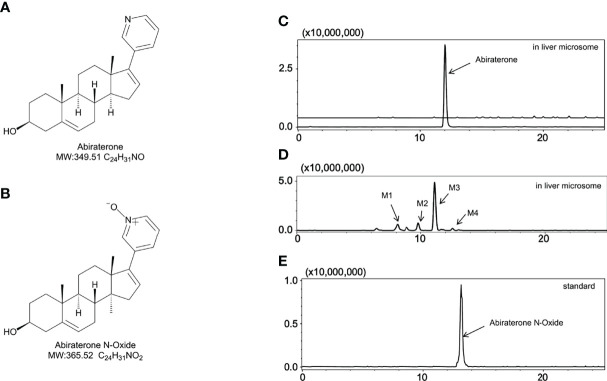
The metabolites of Abiraterone from liver microsomes. **(A)** The chemical structure of Abiraterone. **(B)** The chemical structure of Abiraterone N-Oxide. **(C)** Extracted ion chromatograms (EICs) of Abiraterone after 0 min of liver microsomal metabolism. **(D)** EICs of metabolites of Abiraterone after 120 min of liver microsomal metabolism. **(E)** EICs of the standard Abiraterone N-Oxide.

Prostate cancer is the second most common cancer and the fifth leading cause of death in men worldwide (1). Patients with advanced prostate cancer are often treated with androgen deprivation therapy (ADT) but eventually progress to metastatic castration resistant prostate cancer (mCRPC) (2). Abiraterone(17-(3-pyridyl) androsta-5,16-dien-3beta-ol, molecular formula: C_24_H₃₁NO, molecular weight: 349.51, chemical structure of Abiraterone is shown in **Figure 1A**) is a potent and irreversible CYP17A1 inhibitor with antiandrogen activity (3). Abiraterone (ABR) inhibits the biosynthesis of androgens and estrogens and is mainly used in combination with prednisone for the treatment of mCRPC patients who have previously received docetaxel-containing chemotherapy (4). With the in-depth study of abiraterone clinical trials in recent years, abiraterone was found to be useful in the treatment of newly diagnosed high-risk metastatic castration-sensitive prostate cancer (5–7). ABR, a steroidal antiandrogen, is insoluble in water, resulting in poor bioavailability (8). Abiraterone acetate (ABA) is a prodrug form of ABR (9). Due to the poor solubility of ABA, it is estimated that its fasting absolute oral bioavailability is less than 10%, so the clinical daily dosage is as high as 1000 mg (10). ABA was rapidly hydrolyzed to ABR *in vivo*, so ABA was not detected in plasma (11–13). Studying the metabolic profile of ABR has important implications for therapeutic drug monitoring (13). Trillions of microbes inhabit the human gut, a complex ecological community of bacteria, viruses, fungi, protists, and archaea that influence normal physiology and disease progression through their metabolic activities and host interactions (14, 15). Gut microbiomes are even called new organs of the human body (16). The intestinal microbiota is very diverse and varies from person to person. The composition and function of the intestinal microbiota are unbalanced and are related to various diseases such as human metabolic diseases, cardiovascular diseases and tumors (17). Intestinal flora is rich in a variety of enzymes related to metabolism and improves the quantity and bioavailability of drugs and metabolites with biological effects in the process of drug biotransformation (18). Intestinal flora participates in various reactions such as catalytic oxidation, reduction, decarboxylation, demethylation, isomerization and ring cleavage, which affect the biological effects of drugs (19). Understanding the gut microbiome could enable us to understand disease in new ways. Multi-omics emerged with the in-depth study of gut microbiota, allowing us to study the relationship between gut microbiota and drugs (20).

ABR is a common oral anticancer drug in clinical practice. Due to its low oral availability, its effect on the intestinal flora after oral administration needs to be further studied. The metabolic transformation of ABR by the gut microbiota may be one of the main reasons for this, and the metabolites produced by ABR may have potential pharmacological activities. Therefore, our study mainly focused on the metabolism of gut microbiota and liver microsomes to explore the effect of liver and gut microbiota on ABR metabolism. In this study, ABR was metabolized by the intestinal flora and liver microsomes of Sprague Dawley (SD) rats, and the ABR metabolites from the liver microsomes of Sprague Dawley rats were compared with those from the gut microbiota by LC/MS^n^-IT-TOF to explain its possible metabolic pathways.

## Materials and Methods

### Instruments and Reagents

Abiraterone (CAS: 154229-19-3; Cat Number: SA5840) was purchased from Solarbio Life Sciences Co., Ltd. (Beijing, China). Abiraterone N-Oxide (CAS: 2378463-76-2; Cat Number: A-8216) was purchased from TLC Pharmaceutical Standards Ltd. (Ontario, Canada). The purity of the compounds was higher than 98% (HPLC). HPLC-grade acetonitrile, and methanol were purchased from Fisher Scientific (Fair Lawn, NJ, USA). Sprague-Dawley (SD) rat liver microsomes were purchased from RILD Research Institute for Liver Diseases (Shanghai) Co. Ltd (Cat Number: WWJW). Qualitative identification of ABR metabolites in gut microbiota and liver microsomes and structural analysis using the LC/MS^n^-IT-TOF system from Shimadzu Corporation (Kyoto, Japan). A small refrigerated high-speed centrifuge (Eppendorf Centrifuge 5424 R) was purchased from Eppendorf (Hamburg, Germany). WH-681 vortex mixer was purchased from Jintan Shenglan Instrument Manufacturing Co., Ltd. (Jintan, China).

### Animals

Six Sprague Dawley (SD) male rats (200-300 g) were purchased from Vital River Laboratory Animal Technology Co., Ltd. (Beijing, China). All animals were housed in a ventilated room with free access to food and water, a 12-h light and 12-h dark cycle. Temperature was maintained at 20–24°C and humidity at 40–60%. Before the experiment, the rats were fasted for 12 h and had free access to water. This study was conducted under the permission and guidance of the Laboratory Animal Ethics Committee of the Chinese Academy of Medical Sciences and Peking Union Medical College (Approval number: 00005407). All steps were performed in accordance with the Organizational Guidelines and Ethical Guidelines of the Laboratory Animal Ethics Committee.

### Determination of Abiraterone by LC/MS^n^-IT-TOF

To identify the metabolites of ABR, an LC/MS^n^-IT-TOF equipped with an ESI source was used. Analytes were separated using Luna C_18_-HST column (50 × 2 mm, 2.5 µm, Phenomenex, USA). The mobile phase consisted of formic acid: water (0.1:100, v/v) (as mobile phase A) and acetonitrile (as mobile phase B). The temperature of the column oven was 40°C, and the flow rate was 0.4 mL/min. Mass spectrometry conditions: ionization mode: ESI source, analysis mode: positive and negative ion mode, nebulization gas flow: 1.5 L/min, CDL temperature: 200°C, heating block temperature: 200°C, detector voltage: 1.75 KV, collision energy: 50%, drying gas pressure: 115 KPa, mass spectrometry primary data acquisition range: *m/z* 100~1000, multi-level data acquisition using automatic mode. The elution gradient conditions (A: B) is shown in **Table 1**.

**Table 1:** The elution gradient conditions (A: B).

Time (min)	Mobile Phase A (%)	Mobile Phase B (%)
0	85	15
3	85	15
10	55	45
20	10	90
20.01	85	15
25	STOP	

### *In Vitro* Incubation of Abiraterone With Gut Microbiota

The colon contents of six Sprague-Dawley (SD) rats were collected after sacrifice, and sterilized anaerobic medium (Solarbio Life Sciences Co., Ltd. (Beijing, China) was added with an *m/v* ratio of 1:20 (g/mL), which was mixed evenly and purged with nitrogen after filtering. The mixture was pre incubated under anaerobic conditions at 37°C for 60 minutes. A methanol solution of ABR (1 mg/ml) was prepared, and 10 μL of this solution was added to a presterilized centrifuge tube (the final concentration of ABR in the system was 10 μg/mL), which was mixed with 990 μL of the preincubated mixture under anaerobic conditions. The drug was incubated with the intestinal microbiota at 37°C for 0, 6, 12 and 24 hours. In addition, negative controls containing heat inactivated intestinal microbiota were incubated with ABR for the same time (24 hours). After the incubation, 3-fold volume (3 mL) of pure methanol solution was added to the incubation system and mixed to stop the reaction and precipitate the protein at 0, 6, 12, 24h. After centrifugation using a small refrigerated high-speed centrifuge at 4°C, 13,400 × g rpm for 10 minutes, 100μL was added to a chromatographic autosampler for LC/MS^n^-IT-TOF analysis.

### *In Vitro* Incubation of Abiraterone With Liver Microsomes

The liver microsome incubation system consisted of the following: 5 μL of Sprague–Dawley rat liver microsomes (20 mg/mL), 2 μL of ABR (10 μg/mL, final concentration in the system was 1 µmol/mL), 20 μL of NADPH and 173 μL of Tris/HCl (0.05 mM, pH = 7.4) in a total volume of 200 μL. Culture in a shaking incubator at 37°C and 800 rpm with oxygen. After the incubation, 3-fold volume (600 μL) of pure methanol solution was added to the incubation system and mixed to stop the reaction and precipitate the protein at 0, 15, 30, 60, 90, and 120 min. After centrifugation using a small refrigerated high-speed centrifuge at 4°C, 13,400 × g rpm for 10 minutes, 100 μL was added to a chromatographic autosampler for LC/MS^n^-IT-TOF analysis.

### Statistical Analysis

Data acquisition and processing were performed with Shimadzu LC-MS Solution (version 5.89, Kyoto, Japan). Two-tailed ANOVA and Student’s t-test were used for statistical analysis with GraphPad Prism Version 9 for macOS (GraphPad Software, San Diego, CA, USA). Data are expressed as the mean ± standard deviation (SD), and p values less than 0.05 were considered statistically significant.

## Results

Through this study we aimed to elucidate whether ABR could interact with gut microbiota or liver microsomes to generate unique metabolic signatures. By comparing gut microbiota with liver microsomal metabolites, we wanted to explore the unique role of ABR in gut microbiota and liver microsomes and investigate possible metabolic pathways. **Figure 1A** shows the molecular structure of ABR. In this study, LC/MS^n^-IT-TOF was used to characterize ABR metabolites from SD rat liver microsomes with those from gut microbiota and propose possible mass cleavage pathways. The retention time of ABR in this method is 12 min.

### Metabolism of Abiraterone in Liver Microsomes

**Figure 2:**
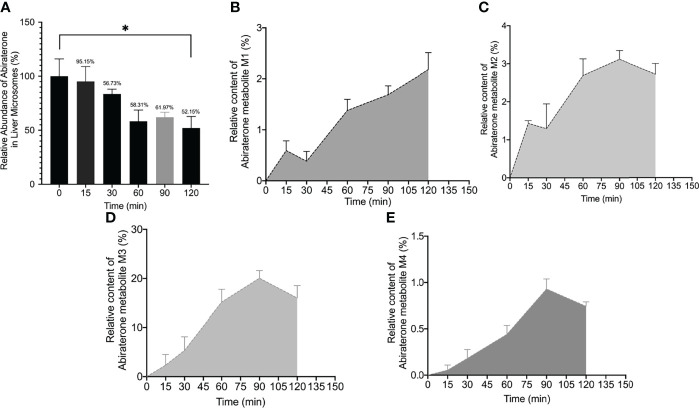
The relative abundance curve of Abiraterone **(A)** and metabolites (M1, M2, M3, M4) **(B–E)** incubated with rat liver microsomes at different time points (0 min, 15 min, 60 min, 90 min and 120 min) (*). *P < 0.05.

To explore the metabolism of ABR in liver microsomes, we performed *in vitro* metabolism experiments using a Sprague-Dawley rat liver microsome incubation system (5 μL of Sprague–Dawley rat liver microsomes + 2 μL of ABR + 20 μL of NADPH + 173 μL of Tris/HCl). The relative abundance of ABR in the Sprague-Dawley rat liver microsome incubation system over time is shown in **Figure 2A**. **Figure 2A** shows that ABR could be metabolized by Sprague-Dawley rat liver microsomes within 120 min.

Interestingly, we found four metabolites (M1, M2, m3 and M4) (In this study, M1-M5 are the abbreviations for metabolites 1-5, which are synonyms for the unknown metabolites of abiraterone found in this study. No further explanation below.) in the liver microsomal incubation system. **Figures 1C**, **D** shows the EIC diagram of metabolites of ABR liver microsome incubated for 0 and 2h. Four hydroxylated metabolites were mainly produced in the metabolism of liver microparticles *in vitro* (the relative abundance changes in metabolism are shown in **Figure 2** BCDE). The possible chemical structures of M1, M2, M3 and M4 are shown in **Figure 4**. The retention times of M1, M2, M3 and M4 are: 8.315, 9.820, 11.045, 12.855 min, respectively. M1~M4 are isomers, ion *m/z* = 366.2597. The MS information of Abiraterone and its metabolites from the liver microparticle incubation system is shown in **Table 2**.

**Table 2:** The multistage mass spectrometry information of Abiraterone and its metabolites from the intestinal microflora metabolic system and liver microparticle incubation system by LC/MS^n^-IT-TOF.

No.	Substance name	Retention time (min)	MS (+)	MS^2^	MS^3^
1	Abiraterone	12.002	350.2601	334.2308 302.2026 156.0879	316.2213 170.0997 144.0805 196.1190
2	M1	8.315	366.2597	334.2327 318.1700 262.1769 207.1398 156.1029	196.1327
3	M2	9.820	366.2567	350.2615 318.2209 156.0858	140.0779
4	M3	11.045	366.2572	350.2303 248.2133 156.0988 120.0569	–
5	M4	12.855	366.2597	334.2191 316.2325 262.2264	–
6	M5	14.763	389.3129	371.3003	325.2909 211.1568 147.1087

### Metabolism of Abiraterone in the Gut Microbiota

**Figure 3:**
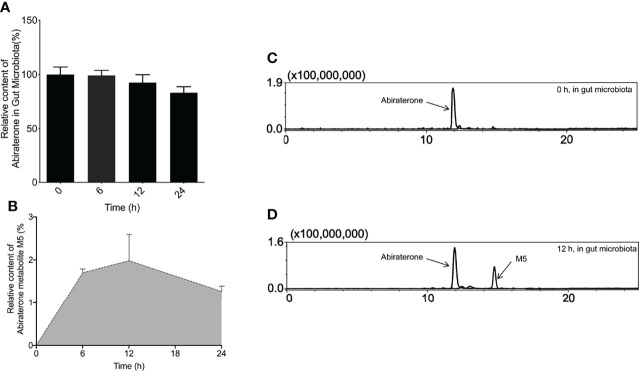
The metabolites of Abiraterone from the gut microbiota. **(A)** The relative abundance of Abiraterone after incubation with rat gut microbiota for 0 h, 6 h, 12 h and 24 h. **(B)** The relative abundance of Abiraterone metabolite M5 after incubation with rat gut microbiota for 0 h, 6 h, 12 h and 24 h. **(C)** EICs of abiraterone after 0 h of incubation in the gut microbiota system. **(D)** EICs of abiraterone and metabolite M5 after 12 h of incubation in the gut microbiota system.

To explore whether the gut microbiota is involved in ABR metabolism, the colonic contents of six Sprague-Dawley (SD) rats were incubated with ABR. At the same time, an incubation system of the colon contents inactivated by heating twice was used as a negative control to eliminate the interference of environmental factors such as the culture medium. After the incubation, 3-fold volume (3 mL) of pure methanol solution was added to the incubation system and mixed to stop the reaction at 0, 6, 12, 24h and prepare samples for detection of ABR content in cultures by LC/MS^n^-IT-TOF. It can be seen from the **Figure 3A** that the content of ABR in the *in vitro* incubation system gradually decreased with time. EIC diagram of ABR and its metabolites metabolized by intestinal flora for 0h and 12h *in vitro* are shown in **Figures 3C, D**. In contrast, heat-killed gut flora hardly metabolized ABR. This suggests that the decline in ABR is the result of co-metabolism by the live microbiota, which demonstrates the role of the gut microbiota in ABR metabolism.

**Figure 4:**
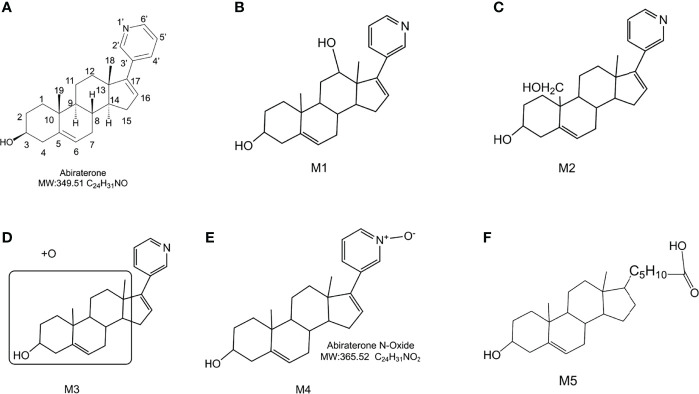
The chemical structure of Abiraterone and possible chemical structures of Abiraterone metabolites. **(A)** The chemical structure of Abiraterone (Abiraterone is numbered according to the IUPAC carbon numbering scheme). **(B)** The possible chemical structure of Abiraterone metabolite M1. **(C)** The possible chemical structure of Abiraterone metabolite M2. **(D)** The possible chemical structure of Abiraterone metabolite M3. **(E)** The chemical structure of Abiraterone metabolite M4 (Abiraterone N-Oxide). **(F)** The possible chemical structure of Abiraterone metabolite M5.

To explore the metabolites of ABR in the intestinal flora, LC/MS^n^-IT-TOF was used to search and infer the structures of the metabolites in the reaction system, and a metabolite(M5) was found (the relative abundance changes in metabolism are shown in **Figure 3B**. EIC diagram of ABR metabolites metabolized by intestinal flora for 12h *in vitro* is shown in **Figure 3D**. Possible chemical structure of M5 is shown in **Figure 4F**). The retention time of M5 is 14.763 min. However, they were not detected in the inactivated gut microbiota system, suggesting that M5 may be a metabolite produced by the gut microbiota metabolizing ABR.

### Structure Analysis of Metabolites

**Figure 5:**
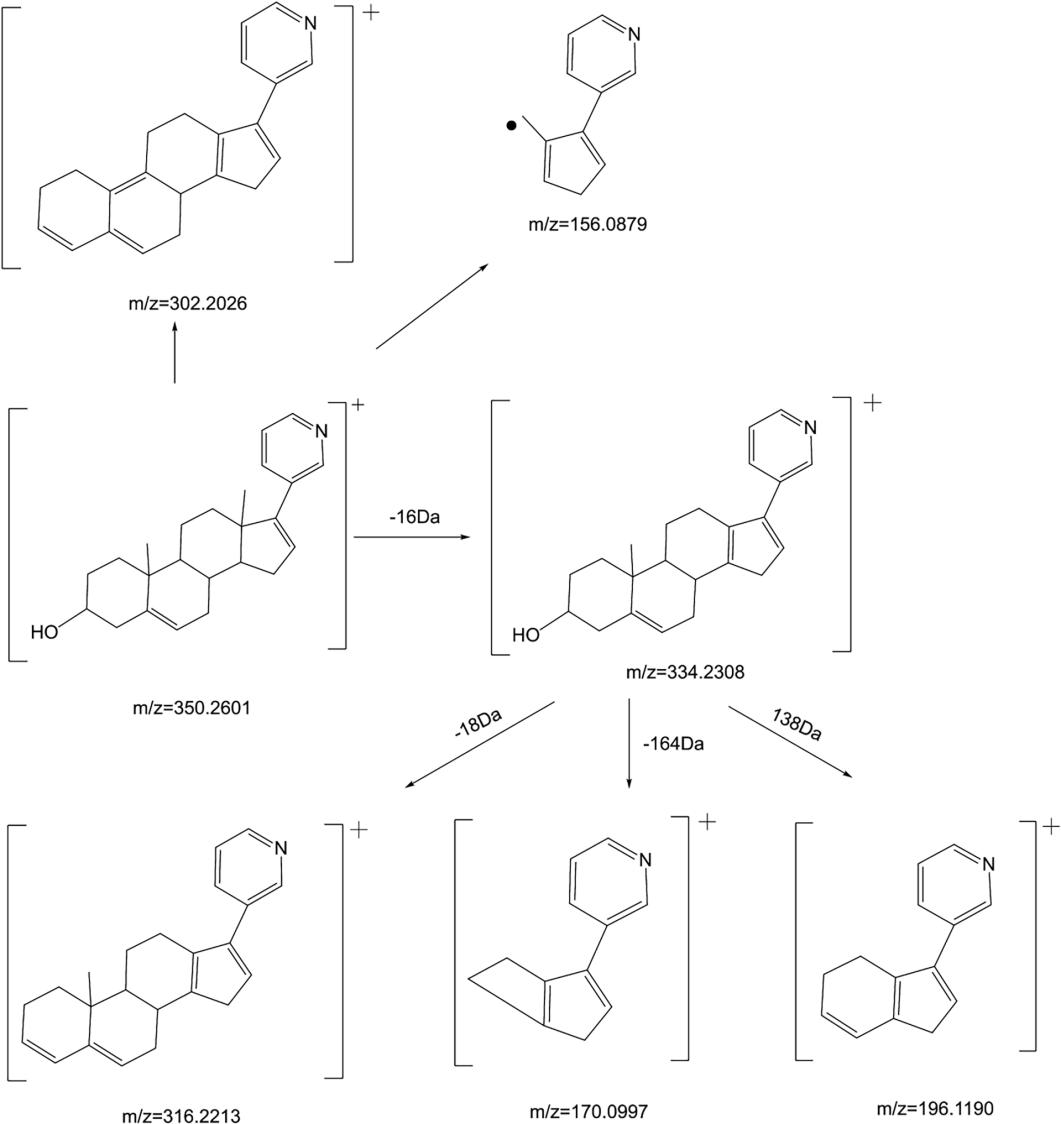
The mass spectrometry fragmentation rule of Abiraterone.

Firstly, we analyze the cracking law of ABR:

ABR [M+H]^+^ is 350.2601, and the secondary fragment ion is 334.2308, which may be obtained from the loss of methane (CH4) on the 10th or 13th carbon (**Figure 4A**). However, the tertiary fragment ion 170.0997 may be obtained from the C8-C14 position and the single bond of C9-C11 is broken. It can also be judged that the secondary fragment is obtained by the neutral loss of 18Da at the 13th carbon, and the tertiary fragment ion 316.2213, which may be the 3rd carbon. dehydration of hydroxyl. The tertiary fragment ion 196.1190 is obtained by further fragmentation of the C9-C10 single bond and the C7-C8 single bond by the secondary fragment ion 334.2308. Secondary fragment ion 302.2026 results from the loss of one water and two methyl groups from parent ion 350.2601. The secondary fragment ion 156.0879 is obtained by the cleavage of the C12-C13 single bond and the C8-C14 single bond. The mass spectrometry fragmentation rule of ABR is shown in **Figure 5**.

The [M+H]^+^ of metabolites M1, M2, M3 and M4 is 366.2573, which are isomers. The molecular weight is 16Da more than that of ABR, suggesting that there is one more oxygen atom in the molecule. These four metabolites were obtained by oxidation reaction.

**Figure 6:**
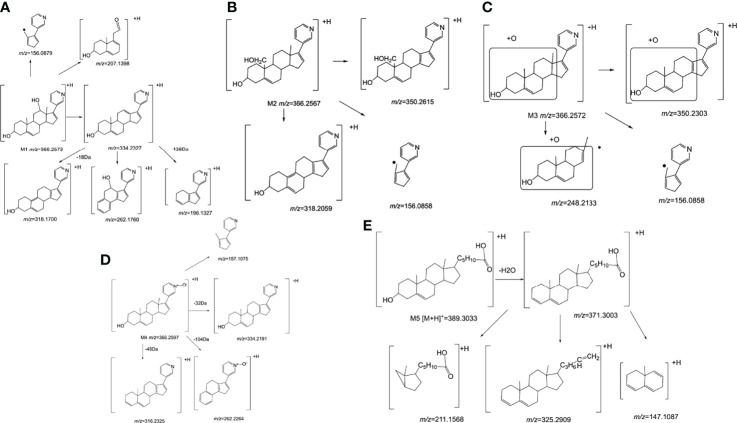
The mass spectrometry fragmentation rule of Abiraterone metabolites. **(A)** The mass spectrometry fragmentation rule of Abiraterone metabolite M1. **(B)** The mass spectrometry fragmentation rule of Abiraterone metabolite M2. **(C)** The mass spectrometry fragmentation rule of Abiraterone metabolite M3. **(D)** The mass spectrometry fragmentation rule of Abiraterone metabolite M4(Abiraterone N-Oxide). **(E)** The mass spectrometry fragmentation rule of Abiraterone metabolite M5.

The [M+H]^+^ of M1 is 366.2573, and the secondary fragment ion 334.2327 is consistent with ABR. Fragments 207.1398 and 156.1029 prove that the oxidation position is not on pyridine ring, five membered ring and C18; The secondary fragment 262.1769 is consistent with the secondary fragment information of M4, indicating that the oxidation position is not on the methyl group, so the possible position of the oxidation position of M1 is C12, C11, C9, C8, C6 or C7. Because the parent ion 366.2573 goes to the secondary fragment ion 334.2317 and loses 32 Da, and the CH_3_OH structure is excluded for the above reasons, then only 1 H_2_O and 1 CH_4_ can be lost at the same time and form a double bond, the parent ion to the secondary 318.1700 loses 48Da, it is speculated that 1 H_2_O and 2 CH_4_ are lost. Therefore, M1 may be oxidized to hydroxyl at C12 or C9, but it is more likely to oxidize at C12 from 262.1769 fragment information. The multistage mass spectrometry information of M1 is shown in **Table 1**. The MS fragmentation rule of M1 is shown in **Figure 6A**. The possible chemical structure of Abiraterone metabolite M1 is shown in **Figure 4B**.

The secondary fragments of M2 and M3 are 350.2615, which may be obtained by the loss of CH_4_ (16Da) by the parent ion. Because M2 and M3 contain fragments 156.0858, it is speculated that the oxidation position is not on the pyridine ring, five membered ring and C18. However, M2 contains the same fragment 318.2209 as M1, which may be that the parent ion 366.2567 loses a CH_4_ and a CH_3_OH, and M2 may be oxidized to methoxy at C19.

There is no 318.2209 in the secondary fragment of M3. The secondary fragment 248.2133 of M3 further indicates that the oxidation position is not on the five membered ring and pyridine ring. The Multistage mass spectrometry information of M2 and M3 are shown in **Table 1**. The MS fragmentation rule of M2 and M3 are shown in **Figures 6B, C**. The possible chemical structure of M2 and M3 are shown in **Figures 4C, D**.

The [M+H] ^+^ of M4 is 366.2597, and the secondary fragment ion is 334.2191, 316.2325, 262.2264. This mass spectrometry information is consistent with Abiraterone N-oxide, a metabolite of ABR in the literature. By comparing the retention time and mass spectrometry information with Abiraterone N-oxide standard samples (The chemical structure of M4 (Abiraterone N-oxide) is shown in **Figure 1B**. The EIC diagram of Standard Abiraterone N-Oxide is shown in **Figure 1E**), we can determine that M4 is Abiraterone N-oxide. The multistage mass spectrometry information of M4 is shown in **Table 1**. The MS fragmentation rule of M4 is shown in **Figure 6D**. The chemical structure of M4 (Abiraterone N-oxide) is shown in **Figures 1B**, **4E**.).

The fragment of intestinal metabolite M5 has great changes with ABR, suggesting that the reaction may be more complex. The [M+H] ^+^ of the metabolite M5 is 389.3033. The molecular weight of M5 is 39.10 Da more than that of ABR. The parent ion 389.3033 to the secondary ion 371.3003 lose an H_2_O, indicating that there is a hydroxyl group in the structure and that the hydroxyl group at the C3 position of ABR remains unchanged. The third-order fragment 325.2909 is obtained from the loss of 46da of fragment ion 371.3003, indicating the possible loss of CO and H_2_O at the same time or the loss of neutral molecular formic acid. Because there is no fragment related to ABR in the fragment, there may be no pyridine ring in the metabolite. The remaining structural molecular weight of ABR without pyridine ring is 274.23, while the [M+H]^+^ of metabolite is 389.3033, with a difference of 115.07. The structural type of ABR is like cholesterol. It is speculated that ABR may lose pyridine ring under the action of intestinal bacteria, connecting a long-chain carboxylic acid will produce a neutral loss of 46 Da, that is, the loss of carboxylic acid. Therefore, the group connected on the five membered ring is C_6_H_12_O_2_. Therefore, it is speculated that the chemical structure of M5 is shown in the **Figure 4F**, and the possible cracking is shown in the **Figure 6E**.

**Figure 7:**
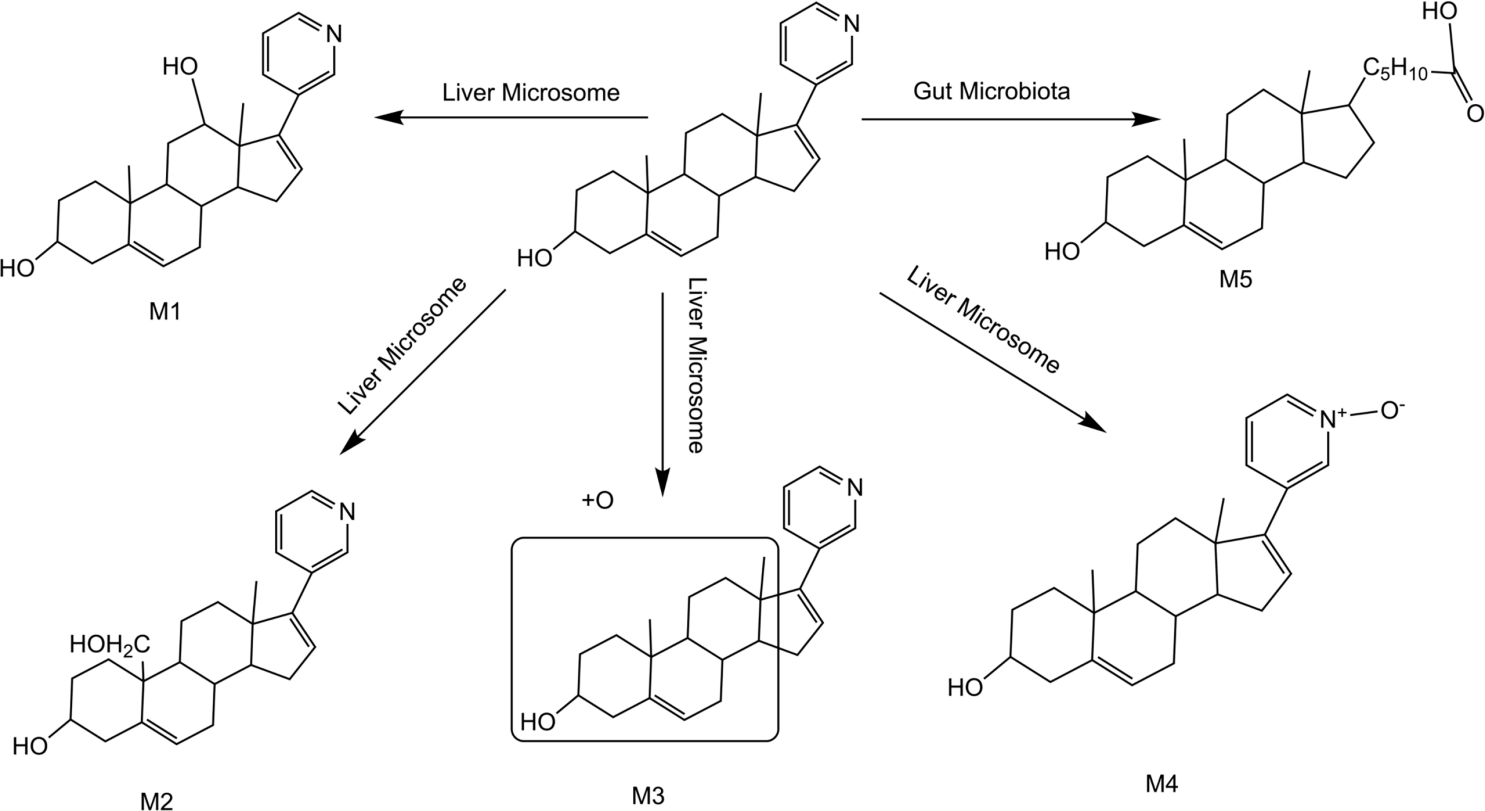
Metabolic pathway of abiraterone in the gut microbiota system and liver microsomes (Abiraterone is metabolized in the gut microbiota to produce M5 and produce M1-4 in liver microsomes).

Summarizing the cleavage laws of the above metabolites, we summarize the metabolic pathway of abiraterone in the gut microbiota system and liver microsomes (Abiraterone is metabolized in the gut microbiota to produce M5 and produce M1-4 in liver microsomes), as shown in **Figure 7**.

## Discussion

Oral antihormonal drugs are essential for the treatment of prostate cancer. The pharmacokinetic exposure of these drugs is known to vary widely among patients, which may lead to underdosing in some patients, resulting in suboptimal efficacy, while overdosing in others may lead to unwanted side effects (21). ABR has low bioavailability after oral administration, so ABA and other prodrug forms are used to make up for the deficiency of the drug itself (22). As a new organ of human body, intestinal flora has attracted more and more attention of scientific researchers. ABR is a commonly used oral antitumor drug, and the gut microbiota may inevitably be involved in its metabolism. In this study, five metabolites were identified from the perspective of intestinal microflora metabolism *in vitro* and combined with the liver microsomal metabolic system of rats. Among them, four metabolites were derived from liver microsomes and one from the intestinal microbiota incubation system. By comparison with the standard sample, we also confirmed that the metabolite from intestinal flora metabolic system was Abiraterone N-oxide, which also reflected that intestinal flora was involved in ABR metabolism. This brings a new research direction for oral anticancer drugs like ABR and provides a theoretical basis for the study of pharmacodynamic substances *in vivo*.

A total of 5 major metabolites were found in this study, among which 4 hydroxylated metabolites were mainly produced in the metabolism of liver microsomes *in vitro*. The cleavage of ABR and each potential degradation product are calculated by combining the information obtained from the analysis of LC/MS^n^-IT-TOF with relevant literature reports (22–24). The [M+H]^+^ of metabolites M1, M2, M3 and M4 is 366.2573, which are isomers. The molecular weight is 16Da higher than that of ABR, suggesting that there is one more oxygen atom in the molecule. These four metabolites are obtained by oxidation reaction. And the order of peaks is M1, M2, M3 and M4, indicating that M1 has a greater polarity. We analyzed their cleavage pathways and derived the possible chemical structures of these four metabolites (shown in **Figures 4**, **6**). However, the physicochemical properties and biological effects of these four metabolites need to be further studied.

It is reported that ABR-sulfate and ABR-sulfate-N-Oxide are the two main circulating metabolites of ABR, accounting for about 43% of the exposure (11, 24, 25). Several methods for bioanalysis of ABR in human plasma have been reported (25, 26). It is well known that oral drugs are absorbed in the gut and undergo phase II metabolism in the liver, where they may be extensively metabolized into glucuronide, sulfate, and glutathione conjugates. However, in this study, phase II metabolites could not be found due to ABR metabolism in liver microsomes *in vitro*. Therefore, the phase II metabolism of ABR *in vivo* needs further study in the future. Interestingly, epoxidation is a recognized pathway for ABR, and epoxidation of steroids has also been described (24). The [M+H]^+^ of M4 is 366.2597, and the secondary fragment ions are 334.2191, 316.2325, 262.2264. This mass spectrometry information is consistent with the metabolite ABR-N-Oxide of ABR in the literature. M4 was confirmed to be ABR-N-oxide by comparing retention time and mass spectral information with ABR-N-Oxide standard. In addition, this study discovered a new metabolite M5 through *in vitro* intestinal flora metabolism experiments.

As described in the Results section, the fragment of intestinal metabolite M5 has great changes with ABR, suggesting that the reaction may be more complex. We analyzed that the structural type of ABR is like that of cholesterol. It is speculated that ABR may lose pyridine ring under the action of intestinal bacteria, connecting a long-chain carboxylic acid will produce a neutral loss of 46 Da, that is, the loss of carboxylic acid. Therefore, the group connected on the five membered ring is C_6_H_12_O_2_. Bile acid is a cholesterol metabolite, which mainly acts on lipid metabolism and has regulatory functions on the whole body. Research on the gut microbiota-bile acid-host axis is expanding into multiple fields including gastroenterology, endocrinology, oncology, and infectious diseases (27). M. Funabashi et al. Elucidating the metabolic pathway of bile acid dehydroxylation in the gut microbiome through microbiome and metabolomics (28). ABR is a steroid drug, and intestinal flora may act on ABR. As mentioned above, ABR loses 46 Da under the action of intestinal flora, which may be carboxylic acid. Therefore, the metabolite M5 produced by ABR through gut microbiota metabolism may follow the gut microbiota-bile acid-host axis and be metabolized through the metabolic pathway of gut microbiome bile acid dehydroxylation, resulting in antitumor activity. However, this hypothesis needs our in-depth study. The metabolism of ABR under intestinal flora may clarify the anti-prostate cancer mechanism of ABR and find more biological characteristics through new ways.

Of course, the limitations of this study should not be avoided. Firstly, we used intestinal contents and liver microsomes from Sprague Dawley (SD) rats. Different species and individuals have different liver metabolic capacity and distribution of enzymes and other media, and there are differences in the composition and distribution of intestinal flora between different species or different individuals of the same species, which may lead to different results. It is therefore necessary to expand the species, and if possible, to validate with human feces and liver microsomes, to expand the significance of this experiment. Secondly, this experiment is limited to *in vitro* research. Therefore, the phase II metabolite of abiraterone could not be found. it is necessary to explore the relationship between phase II metabolism and phase I metabolism of ABR through *in vivo* experiments. In this way, the conclusion will be more reliable. Finally, the physicochemical properties and biological effects of the five metabolites obtained are temporarily unknown. Therefore, in the future, we plan to further extract and prepare the discovered metabolites and verify the physicochemical properties and biological effects of abiraterone metabolites through animal experiments and other methods. These three points are the direction of our team’s next research.

ABR has low bioavailability and achieves its biological effects under the action of liver and gut microbiota after oral administration. However, it is difficult to elucidate its mechanism using conventional techniques. In this study, the dual pathway research model based on gut microbiota and liver microsomes was used for the first time to comprehensively and systematically study the metabolism of gut microbiota and liver metabolism to explore the metabolites of the anti-prostate cancer drug ABR, which is a beneficial exploration of the research model of the mechanism of oral difficult absorbable drugs. With the further study on the biological activity of metabolites of gut microbiota, we believe that abiraterone’s pharmacodynamic mechanism under gut microbiota is expected to be explored and provide new ideas for clinical medication.

## Data Availability Statement

The original contributions presented in the study are included in the article/supplementary material. Further inquiries can be directed to the corresponding author.

## Ethics Statement

The animal study was conducted according to the guide- lines of the Declaration of Helsinki, and the ethics of both institutional guidelines and Chinese Council on Animal Care. And it has been approved by the Laboratory Institutional Animal Care and Use Committees of the Chinese Academy of Medical Sciences and Peking Union Medical College.

## Author contributions

N-ZX conceived the task. AK performed the review and collected original studies. AK, F-YY, WW, S-JH and G-SY wrote the first draft of the manuscript. AK and N-ZX revised the manuscript. N-ZX contributed to language editing and final revision. All data were generated in house, and no paper mill was used. All authors agree to be accountable for all aspects of the work ensuring integrity and accuracy. All authors contributed to the article and approved the submitted version.

## Funding

This project was supported by the CAMS Initiative for Innovative Medicine (No. 2021-I2M-1-015), National Natural Science Foundation of China (No. 81972400). Beijing Hope Run Special Fund of Cancer Foundation of China (No. LC2019B02), Beijing Excellent Talents Program-Youth Backbone Project (No. 2018000032600G393).

## Conflict of Interest

The authors declare that the research was conducted in the absence of any commercial or financial relationships that could be construed as a potential conflict of interest.

## Publisher’s Note

All claims expressed in this article are solely those of the authors and do not necessarily represent those of their affiliated organizations, or those of the publisher, the editors and the reviewers. Any product that may be evaluated in this article, or claim that may be made by its manufacturer, is not guaranteed or endorsed by the publisher.
